# Mild Severe Acute Respiratory Syndrome

**DOI:** 10.3201/eid0909.030461

**Published:** 2003-09

**Authors:** Gang Li, Zhixin Zhao, Lubiao Chen, Yihua Zhou

**Affiliations:** Sun Yat Sen University, Guangzhou, Guangdong Province, China

**To the Editor**: Severe acute respiratory syndrome (SARS) is a recently recognized infectious disease caused by a novel human coronavirus (SARS-CoV) (*1*). The first case of SARS, diagnosed as communicable atypical pneumonia, occurred in Guangdong Province, China, in November 2002. Thousands of patients with SARS have been reported in over 30 countries and districts since February 2003.

SARS is clinically characterized by fever, dry cough, myalgia, dyspnea, lymphopenia, and abnormal chest radiograph results (*1*–*3*). According to the World Health Organization (WHO) (*4*), the criteria to define a suspected case of SARS include fever (>38°C), respiratory symptoms, and possible exposure during 10 days before the onset of symptoms; a probable case is defined as a suspected case with chest radiographic findings of pneumonia and other positive evidence.

Although most reported patients with SARS met the WHO criteria, we found two SARS case-patients who did not exhibit typical clinical features. Case 1 was in a 28-year-old physician. He had close contact with three SARS patients on February 1, 2003. After 10 days, he had mild myalgia and malaise with a fever of 37.3°C. He had no cough and no other symptoms. Leukocyte and lymphocyte counts were normal. The chest radiograph showed no abnormalities. He did not receive any treatment except rest at home. His symptoms disappeared after 2 days. He completely recovered and returned to work 4 days after onset of symptoms. After 12 weeks, his serum was positive for immunoglobulin (Ig) G against SARS-CoV in an indirect enzyme-linked immunosorbent assay (ELISA) with inactivated intact SARS-CoV as the coated antigen.

Case 2 was in a 13-year-old boy whose mother had been confirmed to have SARS on February 4, 2003. Fever developed in the boy 20 days after his mother’s onset of the disease. He did not come into contact with other confirmed SARS patients during this period. He had a mild headache and diarrhea with a fever from 37.2°C to 37.8°C for 3 days. No other symptoms and signs developed, and a chest radiograph showed no abnormalities. He completely recovered after 5 days. After 12 weeks, his serum was positive for IgG against SARS-CoV, detected with an ELISA.

In both case-patients, SARS had been initially excluded in spite of their close contacts with SARS patients because their symptoms could be explained as a common cold, and no specific diagnostic approaches were considered when they were sick since the causative agent of SARS was not identified until March 2003 (*5*). However, their serum specimens were positive for IgG against SARS-CoV by ELISA. Those results strongly indicate that both patients had been infected with SARS-CoV, although their signs and symptoms did not meet the criteria for the SARS case definition. Mild SARS-CoV infection may not easily be defined clinically, and such patients may potentially spread the disease if they are not isolated.
